# Ecofriendly synthesis of cellulose-silver nanocomposites and the evaluation of their antibacterial activity

**DOI:** 10.1186/s11671-024-04156-9

**Published:** 2025-03-28

**Authors:** Ibtissam Charti, Said Sair, Oussama Rafik, Younes Abboud, Abdeslam El Bouari

**Affiliations:** 1 Laboratory of Physical Chemistry, Materials and Catalysis, Faculty of Sciences Ben M’Sick, University Hassan II, Casablanca, Morocco; 2 MAScIR Foundation, VARENA Center, Mohammed VI Polytechnic University, (UM6P) Lot 660, Hay Moulay Rachid, Ben Guerir, Morocco; 3 Department of Biology, Laboratory of Ecology and Environment, Microbiology Unit, Faculty of Sciences Ben M’sik, Hassan II University, Casablanca, Morocco

**Keywords:** Silver nanoparticles, Cellulose-silver nanocomposite, Antimicrobial activity

## Abstract

The integration of nanotechnology with cellulose matrices has gained considerable attention due to the resulting enhanced mechanical, thermal, and antibacterial properties. This study introduces a facile and environment-friendly microwave-assisted method for synthesizing cellulose/Ag nanocomposites. Palm date wood extract was used as an efficient reductant for silver ions, facilitating their deposition onto cellulose surface. The cellulose-silver nanocomposite was synthesized by reducing silver in situ on the surface of cellulose extracted from date palm wood fibers. The extraction involved a series of specific chemical treatments, including alkalization and whitening. The resulting nanocomposite was subjected to various characterization techniques. FTIR spectra showed the elimination of non-cellulosic components post chemical treatments, while XRD affirmed the presence of cellulose peaks. Experimental results indicated that the palm date wood extract was an effective reductant for silver ions favoring the formation of silver with higher crystallinity and mass content in the nanocomposites. Silver nanoparticles were identified within the cellulose matrix through Scanning Electron Microscopy (SEM). The FTIR spectral characterization studies demonstrated the existence of silver in the cellulose nanocomposites. Additionally, the XRD analysis confirmed the formation of silver peaks within these composites. Qualitative antibacterial tests towards gram negative (Escherichia coli) and gram positive (Micrococcus luteus) bacteria were carried out and the results showed that the Ag-MFCs effectively inhibit the growth of both types of bacteria, with 9–13 mm of inhibition zone for both the bacteria. The ecofriendly synthesis method using cellulose as a stabilizing agent proved to be effective in producing well-dispersed spherical AgNPs. The synthesized cellulose silver nanocomposite demonstrated notable antibacterial properties, indicating their potential for applications in medical and environmental fields. This study highlights the feasibility of using green synthesis methods to develop nanocomposites with significant antibacterial activity.

## Introduction

Researchers are actively working on the development of innovative materials sourced from renewable and environmentally sustainable resources to generate valuable products. Over the past few decades, materials based on polysaccharides have emerged as highly promising options due to their abundant availability and ecological viability. Furthermore, their utilization across diverse fields has witnessed a rapid growth.

Polysaccharides, including cellulose, chitosan, starch, and alginate, represent exceptional instances of eco-friendly polymeric materials that find applications across various industrial and biomedical domains.

Cellulose, as a biopolymer abundant in nature, has garnered significant attention in recent years for its versatile applications in various fields including biomedical, environmental, and industrial sectors [1]. Its exceptional properties such as biocompatibility, biodegradability, and mechanical strength make it an attractive candidate for composite materials [2]. Moreover, its cellulosic structure contains numerous functional groups, making it easy to modify or chemically graft, resulting in the creation of novel materials suitable for various applications and make it an attractive candidate for composite materials [3, 4]. In particular, the incorporation of metallic nanoparticles into cellulose matrices has emerged as a promising strategy to engineer materials with enhanced functionalities [5, 6].

Among metallic nanoparticles, silver nanoparticles (AgNPs) have received considerable interest due to their unique physicochemical properties, including high surface area-to-volume ratio and excellent antimicrobial activity [7–9]. Integration of AgNPs into cellulose matrices not only capitalizes on the inherent properties of cellulose but also imparts additional functionalities, such as antimicrobial, catalytic, and optical properties, to the resulting nanocomposites [10, 11]. Silver nanoparticles are one of the most widely studied metal nanoparticles due to their excellent antimicrobial properties. When incorporated into cellulose matrices, they have shown promising results in inhibiting the growth of a wide range of microorganisms, including bacteria, fungi, and viruses. Cellulose-silver nanocomposites, combining the biocompatibility of cellulose with the broad-spectrum antibacterial properties of silver nanoparticles, are highly promising in healthcare. They are effective in wound dressings [12, 13], medical textiles [14], implant coatings, and drug delivery systems due to their antimicrobial activity, enhanced mechanical properties, and biodegradability. These nanocomposites can help reduce hospital-acquired infections, improve wound healing, and enhance the durability of medical devices.

In addition to direct healthcare applications, silver cellulose composites can be employed in water purification systems within healthcare facilities [15]. These composites can effectively remove or inactivate pathogenic microorganisms present in water sources, ensuring the safety of water used for patient care, drinking, and sanitation purposes. Furthermore, eellulose-silver nanocomposites represent a significant advancement in food packaging materials [1]. Their ecofriendly synthesis, combined with effective antimicrobial properties and biodegradability, makes them ideal candidates for improving food safety and reducing environmental impact.

However, challenges such as potential cytotoxicity, regulatory hurdles, and environmental impact need to be addressed to ensure their safe and effective use in healthcare settings.

In this context, the synthesis of cellulose-silver nanocomposites has been a subject of extensive research. Various methods have been explored to achieve the uniform dispersion of AgNPs within cellulose matrices, including chemical reduction, in situ synthesis, and electrospinning techniques [16–18]. In recent years, significant advancements have been made in the field of in situ synthesis of cellulose-silver nanocomposites, driven by the demand for sustainable and functional materials with tailored properties [19].

The integration of silver nanoparticles (AgNPs) into cellulose matrices via in situ synthesis has emerged as a promising strategy for the fabrication of nanocomposite materials with enhanced properties and functionalities [19].

In situ synthesis involves the simultaneous formation of AgNPs within the cellulose matrix, resulting in intimate contact between the nanoparticles and the cellulose fibers, which can lead to improved dispersion and interfacial interactions, ultimately influencing the properties of the resulting nanocomposites. It has been reported that this technique is the most common method for the preparation of silver/cellulose composites. [19, 20].

For example, the in situ synthesis of AgNPs in cellulose fibers in the presence of sodium borohydride as a reducing agent was studied by Zhu et al., 2009. It was found that the sizes of the formed AgNPs change with the change of NaBH4 concentrations [21]

Alternative reducing agents like ethylene glycol (EG), glucose, and ascorbic acid, known for their non-toxic nature, are frequently preferred in the synthesis of cellulose/Ag nanocomposites through a microwave-assisted approach. This method replaces organic solvents and hazardous chemicals, which pose environmental risks, during the preparation of AgNPs nanocomposites. However, this substitution significantly constrains their practical use in biomedical applications [22–24]. To enhance the environmental sustainability of AgNPs production, researchers investigated the use of various natural substances, such as plant extracts, in the in situ synthesis of silver nanoparticles.

Thus, in a previous study, the authors utilized Ocimum sanctum leaf extract as additional reducing agents within a cellulose matrix to produce AgNPs [25] and copper nanoparticles (CuNPs) [26] as well as Terminalia catappa leaf extract in the synthesis of CuNPs [26]. L. Muthulakshmi prepared a cellulose/AgNPs composite films with in situ generated AgNPs by a simple method at ambient conditions using T. cattapa leaf extract as a reducing agent [16].

Furthermore, Microwave heating has garnered significant interest as an innovative approach for the one-step creation of metallic nanostructures in liquid solutions. Utilizing microwaves for this purpose represents a practical and eco-friendly route for producing nanomaterials, offering various attractive advantages, including accelerated reaction rates, shortened reaction durations, decreased energy usage, and environmental sustainability [27, 28]. Moreover, microwave heating consistently yields nanostructures that are smaller in size, have narrower size distributions, and exhibit a greater degree of crystallization when compared to traditional methods like using an oil bath [28].

In our study, we employed date palm wood as both as source of cellulose and phytochemical elements for the reduction process, aiming to synthesize eco-friendly cellulose silver nanoparticles in a cost-effective manner.

Hence, in this study, we reported a facile microwave-assisted synthesis of cellulose–silver nanocomposites using microcrystalline cellulose extracted from date plam wood fiber. The Ag nanoparticles were formed in situ on the surface of cellulose. Date palm wood extract is used as a reducing reagent; thus, no additional reductant was needed. The current approach introduces a novel method for preparing cellulose/Ag nanocomposites without relying on any chemical reducing agents or toxic solvents. The cellulose nanocomposite (MFC-Ag) was characterized by Fourier transform infrared (FT-IR) spectroscopy, scanning electron microscopy (SEM), and X-ray diffraction (XRD). The main aim of the present work was to fabricate antibacterial materials utilizing in situ generated silver nanoparticles (AgNPs) through a simple and cost-effective method using date palm wood extract as a reducing agent for medical applications such as surgical aprons, wound healing and wound cleaning materials, hospital bed spreads etc.

## Methods

### Cellulose extraction

The extraction of cellulose fibers (bleached cellulose) from natural fibers obtained from date palm wood (DPW) is a crucial step for the potential production of microfibrillated cellulose (MFC). To achieve this, a three-step cellulose extraction process was employed, involving a water treatment step, an alkaline treatment step and a bleaching step.

### Water treatment

The water treatment of DPW is a highly important step. On one hand, it removes dirt and impurities from the fiber surface that could hinder direct contact between the alkaline solution and the fibers in the subsequent step. On the other hand, it enhances the efficiency of the subsequent chemical treatments. This treatment initially involves treating the ground DPW fibers with water for one hour at a temperature of 60 °C under mechanical agitation. In this treatment, the solid-to-liquid ratio was set at 1 g/25 ml. The fibers are then filtered and washed with distilled water.

### Alkaline treatment

The alkaline treatment affects the chemical composition, degree of polymerization, and molecular orientation of cellulose crystallites due to the removal of lignin, residual hemicellulose, and other water-insoluble materials. To extract pure cellulose and remove non-cellulosic components, the DPW fibers previously treated with water were immersed in a 4% mass NaOH alkaline solution at a temperature of 80 °C for 2 h under mechanical agitation. This procedure is repeated three times. In this treatment, the solid-to-liquid ratio is 1 g/25 ml. After each treatment, the fibers were filtered and washed with distilled water. This treatment results in fibers with a yellowish color, and the obtained sample is referred to as CBPDT (Treated Date Palm Wood Cellulose). During this treatment, the soda attacks the various hydroxyl groups in the fiber structure, leading to their cleavage and formation of alkoxides.

### Bleaching treatment

Among the most challenging polymers to remove from lignocellulosic fibers is lignin, as its residual presence would hinder the extraction of MFC from bleached cellulose. In this regard, the bleaching treatment is a crucial step to eliminate the organic deposits from the walls, thereby obtaining pure cellulose. To achieve this, DPW fibers previously treated with NaOH (BPDT) were bleached four times under magnetic agitation at a temperature of 80 °C for 4 h using a solution consisting of a mixture of 1.7% (w/v) sodium chlorite solution and acetate buffer solution.

Between each bleaching cycle, the fibers were thoroughly washed with distilled water. The purified cellulose fibers were either dried and stored or suspended in water, which was kept in the refrigerator after adding a few drops of chloroform to prevent bacterial growth. This bleaching treatment results in pure white cellulose fibers or bleached white cellulose, and the sample is referred to as "CB" (Bleached Cellulose) in the subsequent study.

### In-situ elaboration of Ag-MFC nanocomposites

**Fig. 1:**
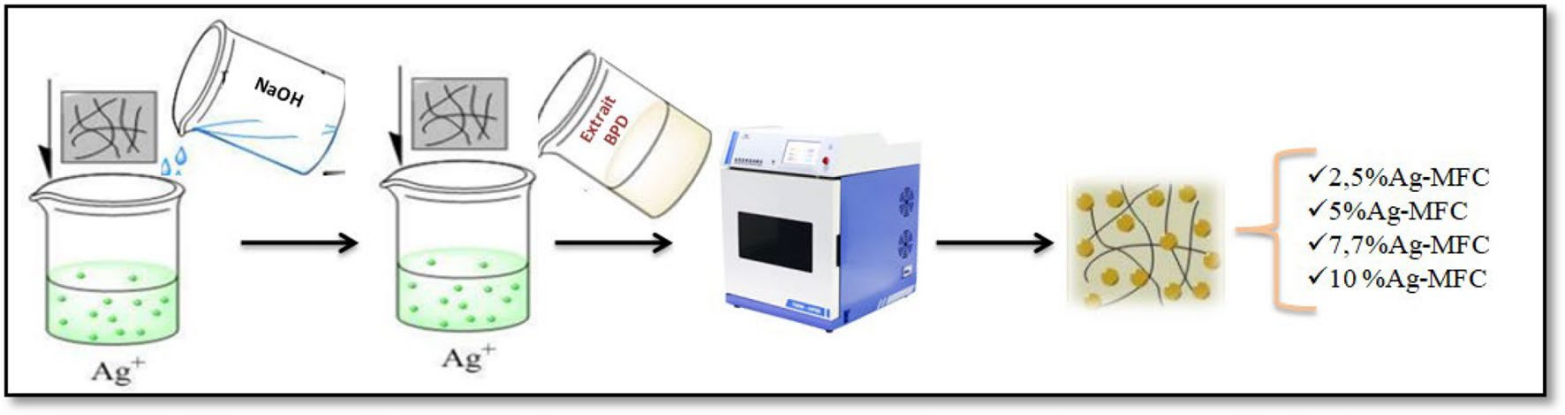
Ag-MFC Nanocomposites Preparation Protocol

Ag-MFC nanocomposites were prepared by in-situ reduction of Ag^+^ ions on the surface of cellulose fibers. The production steps for these nanocomposites are illustrated in Fig. 1. An alcoholic solution of silver nitrate was mixed with a suspension of MFC (12% by weight) under vigorous agitation. Subsequently, a sodium hydroxide solution (0.1 mol/L) was carefully added dropwise to the mixture under vigorous agitation until a pH > 10 was maintained. Immediately afterward, the reducing agent extracted from date palm trees (2 g/25 ml of water) was added to the reaction mixture. The reaction continued for 40 s under microwave power of 400 W until a brown-colored suspension was observed. The obtained Ag-MFC was separated using a centrifuge at 3000 rpm and washed three times with distilled water. Finally, the collected material was dried at 100 °C overnight. Four nanocomposites impregnated with varying concentrations of silver nanoparticles were prepared following the same protocol described in Fig. 1. The quantities used for each constituent are summarized in Table 1.

**Table 1:** Quantity of reagent used for the production of nanocomposites

	MFC (g)	AgNO_3_ (mg)	Agent réducteur (mL)
2,5% Ag-MFC	2	32	50
5% Ag-MFC	2	64	50
7,5% Ag-MFC	2	96	50
10% Ag-MFC	2	160	50

### Antibacterial activity

The antibacterial activity of our nanocomposites was studied in vitro using the method of diffusion of disks. Two bacterial microorganisms were used in this experiment: Micrococcus luteus ATCC9341 (Gram +) and Escherichia coli ATCC25922 (Gram-). The tested microorganisms were individually cultured on sterilized Muller-Hinton Agar (MHA) at 37 °C for 24 h. A colony was sampled using a flamed wire-loop, and the growth was then transferred into a sterilized test tube containing 5 ml of sterilized normal saline solution. The test tubes that contain the bacterial suspension were vortexed to be mixed well uniformly. Then, the bacterial suspension was adjusted to 0.5 McFarland turbidity. A volume of bacterial suspensions was poured into each MHA plate and it was spread on the agar plate surface. Then the disks containing the antibiotic and the disks of different concentration of the MFC-Ag composite were deposited, and a pastille of cellulose MFC was deposed as a negative control. The Plates were incubated at 37 °C for 24 h. At the end of the incubation, the activity of the composite was evaluated by measuring the diameter (mm) of the circular zone transparent around the disks, which corresponds to the absence of bacterial growth.

### Characterization

FTIR spectral measurements were conducted on Bruker Tensor 27 spectrophotometer (Ettlingen, Germany). AgNPs powders were mixed with KBr then pressed to form pellets. The spectra were recorded for wavenumber from 4000 to 400 ​cm^−1^ employing a total of 100 scans with a resolution of 2 ​cm^−1^.

The X-ray diffraction (XRD) analysis was performed using Bruker D8 Advance powder X-ray diffractometer with (CuKα) radiation (45 ​kV, 40 ​mA) and at angular ranging from 10° < 2θ ​ < ​90°. The X-ray diffraction pattern has been identified using the X'Pert HighScore software and ICOD database.

The scanning electron microscopy (SEM) technique produces three-dimensional images of sample surfaces with a resolution that can reach a few nanometers (nm) and a very large depth of field. We used a Hirox SH4000M scanning electron microscope coupled with microanalysis (detector 133 eV). Solid samples were affixed on copper stubs using double-sided adhesive tape, and then a thin layer of carbon is deposited under vacuum conditions to prevent surface charging phenomena for non-conductive samples.

## Results and discussion

### MFC

**Fig. 2:**
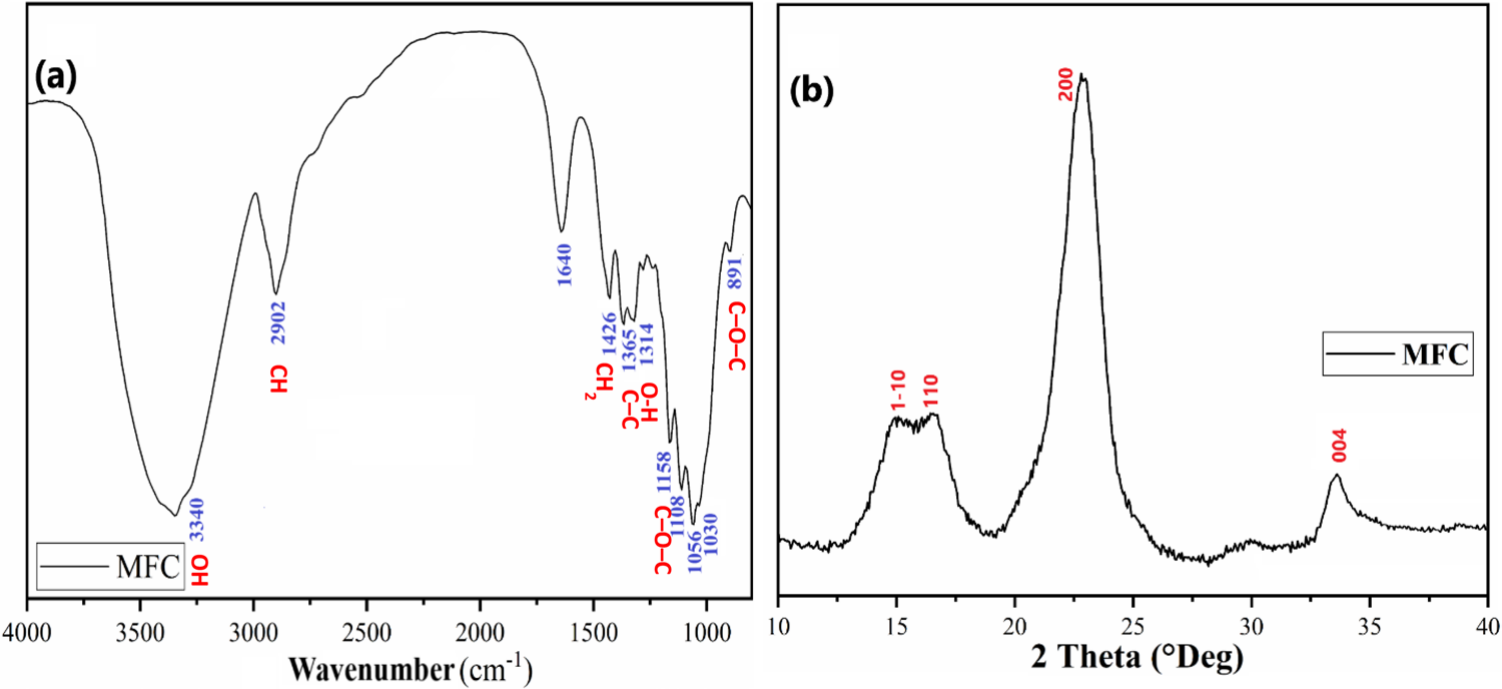
FTIR spectra **a** and X-ray diffractograms **b** of cellulose microfibers

To ensure the successful extraction and preparation of cellulose microfibrils (MFC), the cellulose powder underwent characterization using Fourier-transform infrared spectroscopy. The FTIR spectrum of MFC (Fig. 2a) exhibits a broad band at 3340 cm^−1^ associated with the symmetric stretching of the OH groups and a low-intensity band at 2902 cm^−1^ linked to C-H stretching [29, 30]. A moderately intense band at 1640 cm^−1^ is attributed to the deformation mode of water adsorbed on the surface of the extracted fibers. Bands characteristic of the cellulose structure, assigned to the stretching mode of C–O–C bonds present in β-(1 → 4)-glycosidic linkages, were recorded at 1158 cm^−1^ and 891 cm^−1^. The observation of these bands confirms the increase in the percentage of cellulose components after the chemical removal of non-cellulosic materials such as lignin [31, 32]. Bands at 1426 and 1365 cm^−1^ are respectively attributed to CH_2_ deformation and O–H, while the band associated with the skeletal vibrations of C–C and C–O in the cellulose structure was recorded at 1314 cm^−1^. No band related to the lignin structure was found, confirming the effective elimination of lignin during the treatment [33]. Similar results were reported by Madivoli et al. [34]. Through the isolation of cellulose nanofibers from Oryza sativa residues.

The cellulose powder was also subjected to X-ray diffraction analysis. The X-ray diffractogram of the extracted cellulose microfibers is depicted in Fig. 2b and reveals the presence of diffraction peaks at 15.15°, 16.32°, 22.73°, and 34.35°, associated respectively with the crystallographic planes of (1–10), (110), and (200). These peaks characterize the cellulose structure.

**Fig. 3:**
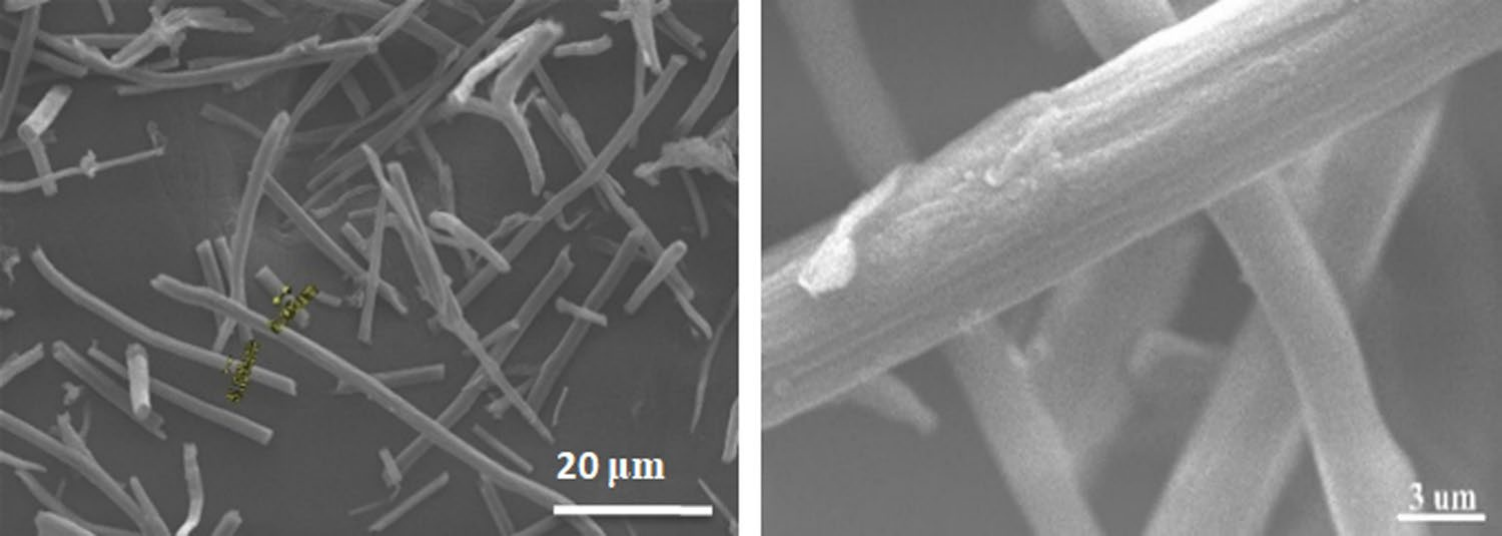
SEM images of isolated cellulose microfibrils (MFC) from date palm wood

In order to determine the morphological aspect of the synthesized cellulose microfibers, observations were made using a scanning electron microscope (SEM) (Fig. 3). The SEM images show well-separated micro-sized fibers with a smooth external surface. The diameter of the synthesized microfibers ranges from approximately 6 to 10 μm. From these SEM images, we can observe the absence of fats, waxes, and any trace of non-cellulosic materials on the surface of the microfibrils, thus concluding that the cellulose microfibrils have been effectively isolated.

### Ag-MFC

**Fig. 4:**
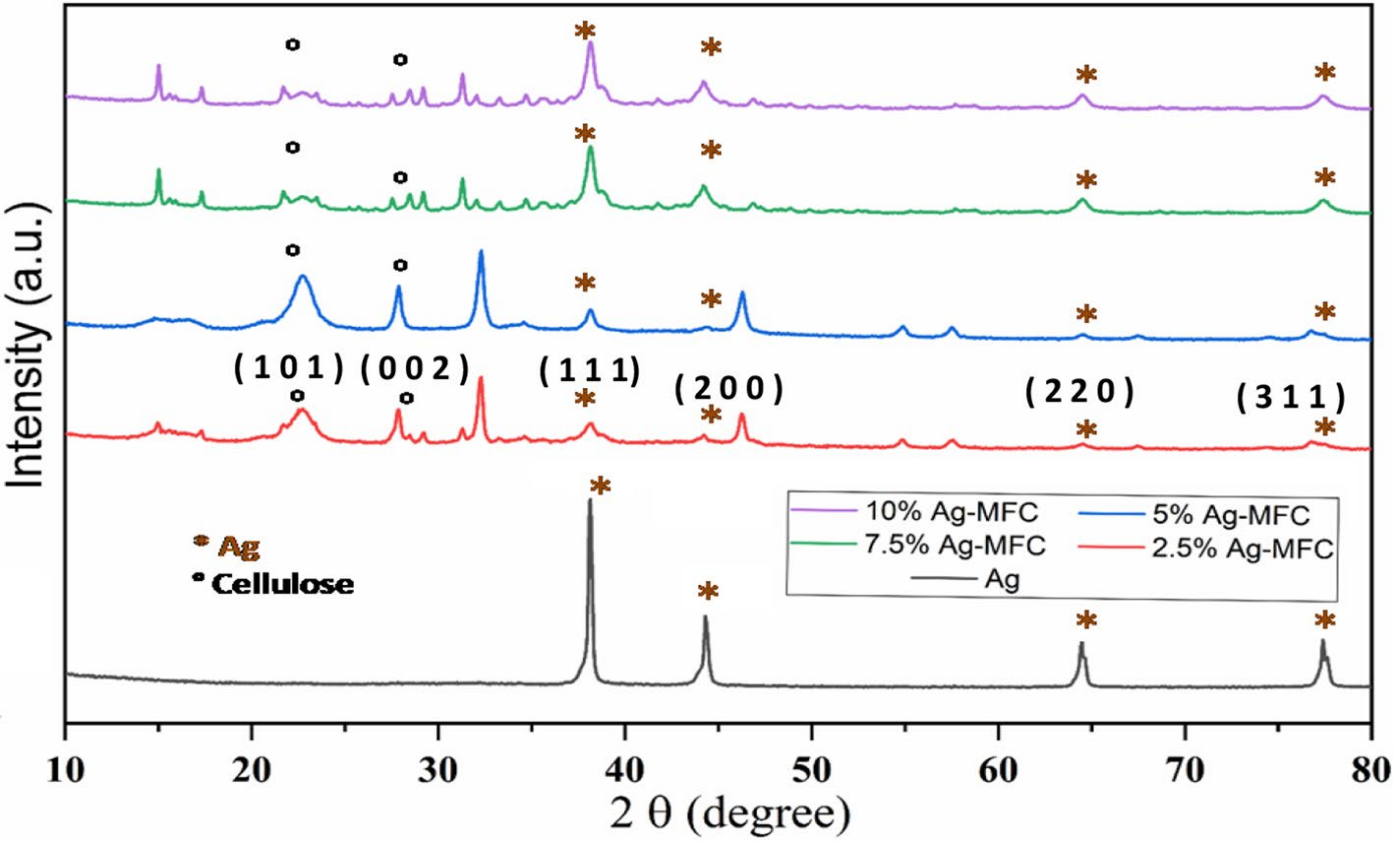
X-ray diffractograms of nanocomposites

In order to confirm the reduction of Ag + ions in the presence of cellulose microfibrils, we conducted X-ray diffraction analyses. The XRD patterns of the nanocomposites (Fig. 4) revealed the presence of four peaks at 2θ values of 38.02°, 43.58°, 64.32°, and 77.22°, which can be attributed to the crystalline planes (111), (200), (220), (311), and (222) corresponding to the existence of pure silver nanoparticles, indicating the successful biosynthesis of AgNPs. Furthermore, distinct peaks at 2θ = 15.4° and 22.7°, corresponding to the crystallographic planes (101) and (002) of the semi-crystalline cellulose microfibril network, were observed for all the nanocomposites. It was noted that the intensity of the AgNPs' diffraction peaks followed the following order: 2.5%–AgNPs-MFC < 5%–AgNPs-MFC < 7.5%–AgNPs-MFC < 10%–AgNPs-MFC, primarily due to the increase in silver percentage in the composites. These results confirm the reduction of silver nanoparticles in the presence of MFC.

**Fig. 5:**
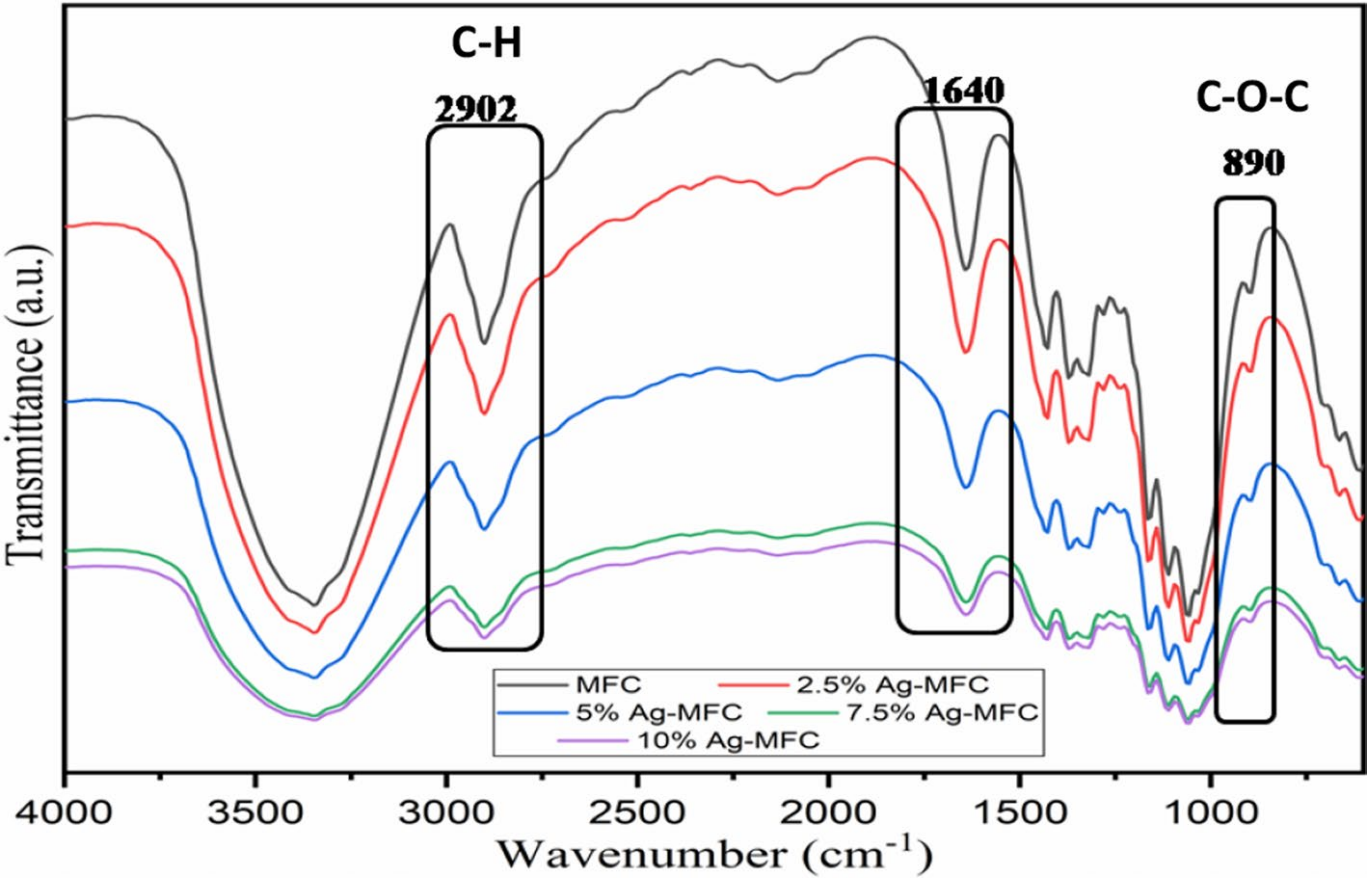
Infrared spectra of Ag-MFC nanocomposites

The FT-IR spectra (Fig. 5) are very similar for all the prepared nanocomposites. From the infrared spectra, the O–H stretching vibration band that appeared at 3350 cm^−1^ in the case of MFC has shifted to 3400 cm^−1^ and become narrower, which could be attributed to the interaction between AgNPs and hydroxyl groups. This behavior has also been reported in subsequent studies [35]. The intensity of the absorption peak at 1426 cm^−1^, assigned to symmetric bending vibrations of CH_2_ groups in the C_6_ of MFC chains, decreased after nanoparticle impregnation. This suggests that some hydroxyl groups in the C_6_ of MFC chains were also involved in the reduction reaction. All these results confirm the presence of biosynthesized AgNPs in the nanocomposites.

**Fig. 6:**
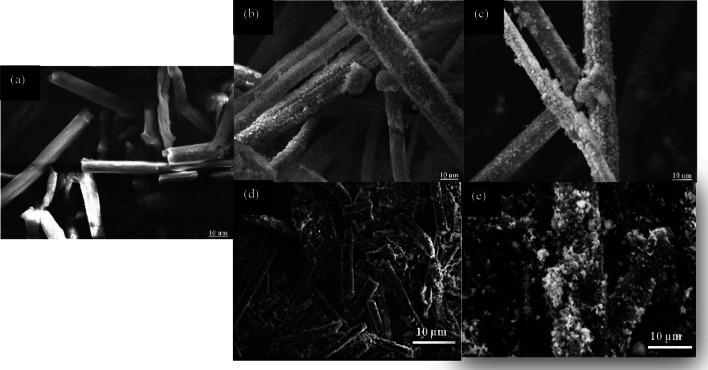
SEM images of MFC (**a**), 2.5%Ag-MFC (**b**), 5%Ag-MFC (**c**), 7.5%Ag-MFC (**d**) and 10%Ag-MFC (**e**)

To verify the formation of AgNPs on MFC, the prepared nanocomposites and MFC were analyzed using scanning electron microscopy (SEM) (Fig. 6).

SEM images of MFC (Fig. 6.a) reveal a uniform, smooth, and clear fibrous structure with an absence of particles across the various fiber levels, Similar results were reported by Muthulakshmi et al.[16], where the AgNPs formed were spherical in shape.

However, the presence of nanoparticles was observed in the case of all the prepared nanocomposites. SEM images in Fig. 6.b and 6.c show uniform nanoparticles that are evenly embedded onto the fiber. Nevertheless, images 6.d and 6.e depict a significant particle agglomeration. The population of deposited AgNPs is much higher in the case of nanocomposites (7.5% Ag-MFC and 10% Ag-MFC). The SEM observations demonstrate that cellulose microfibrils have served as an effective scaffold for introducing metallic ions. Silver ions aggregate through chemical reduction, resulting in immobilized AgNPs on cellulose fibers with high-density growth 2,5%Ag-MFC, 5%Ag-MFC 7.5%Ag-MFC, and 10%Ag-MFC, respectively.

### Antibacterial activity

**Fig. 7:**
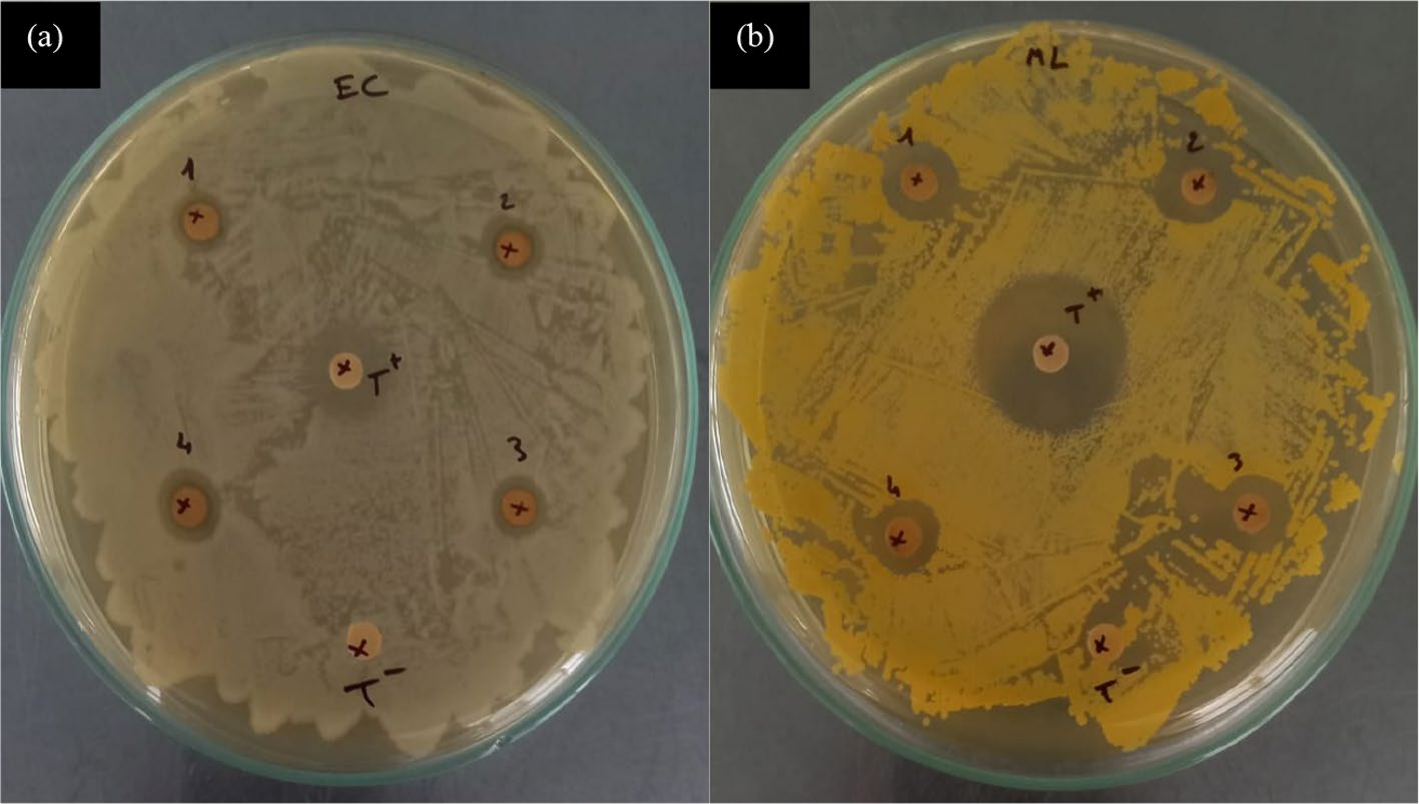
Antibacterial assay: zones of inhibition against **a** Escherichia coli, **b** Micrococcus luteus strain 2.5%Ag-MFC (1), 5%Ag-MFC (2), 7.5%Ag-MFC (3) and 10%Ag-MFC (4) nanocomposites

The biosynthesized nanocomposite demonstrate potent antibacterial effects against both gram-negative and gram-positive bacteria, including multidrug-resistant strains. The antibacterial efficacy of the prepared nanocomposite against various human pathogens was assessed, with the results presented in Fig. 7. Furthermore, Gram-negative bacteria E. coli exhibited inhibition zones with diameters of 9–13 mm. These results are comparable to standard antibiotics (chlortetracycline) (Table 2) similar results were founding by Deeshka et al.[36] by nanocomposite fabrics with in situ generated silver nanoparticles by bioreduction method. To elucidate the specific contribution of the extract molecule in the antibacterial activity against the tested bacteria, bacteriological cavities containing the MFC were examined. The results indicated a complete absence of any appreciable inhibition zone, confirming that the antibacterial activity was primarily caused by the AgNPs alone, without any synergistic effects.

**Table 2:** Inhibition zone diameter (mm) obtained by diffusion test against E. coli and M. luteus strain

	Micrococcus Luteus			Escherichia Coli		
	1st	2nd	3rd	1st	2nd	3rd
0.1	12	12	13	8	9	9
0.25	13	12	13	9	10	9
0.5	11	11	12	10	11	10
0.75	12	11	12	9	9	9
ATB	24	23	23	14	15	14

It is observed that the inhibition zone increases with the increase in the concentration of silver nanoparticles on the surface of cellulose, up to the concentration of 7.5 where we notice that the inhibition zone begins to decrease. This is due to the effect of agglomeration of silver nanoparticles introduced previously as shown in the SEM images.

## Conclusion

An effective and environmentally friendly microwave-assisted method was developed for synthesizing cellulose/Ag nanocomposites. XRD analysis confirmed the face centered cubic (fcc) structure of the silver nanoparticles. FTIR spectroscopy revealed that during the synthesis of cellulose/Ag nanocomposites, there were no significant structural changes observed in the cellulose fiber.

SEM imaging illustrated an increase in the distribution of silver nanoparticles on the cellulose fiber surface, accompanied by a decrease in the utilization rate of silver ions with higher doses of AgNO_3_.

The cellulose/silver composite exhibited good antibacterial properties against M.luteus and E. coli strain.

## Data Availability

The data that support the findings of this study are available on request from the corresponding author. All relevant data are within the manuscript and its Additional files.

## Abbreviations

AgNPs: Silver nanoparticles
DPW: Date palm wood
MFC: Cellulose microfibrils
Ag-MFC: Cellulose-silver nanocomposite
CBPDT: Treated Date Palm Wood Cellulose
CB: Bleached Cellulose
E. coli: Escherichia coli
M. luteus: Micrococcus luteus
